# Comparative insights into structural and optical properties of ZnO/Ag/ZnO and Ag/ZnO/Ag ternary layer thin films

**DOI:** 10.1039/d5na00629e

**Published:** 2025-10-21

**Authors:** S. S. Fouad, L. I. Soliman, E. Baradács, N. F. Osman, M. Nabil, M. E. Sayed, János J. Tomán, Neeraj Mehta, Zoltán Erdélyi

**Affiliations:** a Department of Physics, Ain Shams University Cairo 11566 Egypt; b Department of Solid-State Physics, National Research Center Cairo Egypt; c Department of Environmental Physics, University of Debrecen, Poroszlay u.6 Debrecen-4026 Hungary zoltan.erdelyi@science.unideb.hu; d Departement of Solid-state Physics, University of Debrecen P. O. Box 400 Debrecen-4002 Hungary; e Basic Science Department, Modern Academy for Engineering and Technology in Maadi Cairo Egypt; f Department of Basic Engineering Sciences, Faculty of Engineering (Shoubra) Benha University Benha Egypt; g Physics Department, Banaras Hindu University Varanasi-221005 India dr_neeraj_mehta@yahoo.co.in

## Abstract

The impact of the Ag layer position in ZnO/Ag/ZnO and Ag/ZnO/Ag ternary thin films (thickness: 50 nm) was studied. ZnO was synthesized using atomic layer deposition, while Ag was deposited *via* direct current (DC) magnetron sputtering. Structural and optical properties were analyzed using GIXRD, SEM, and elemental mapping. Key parameters, including thickness, mass density, crystallite size, dislocation density, and surface roughness, were evaluated. Optical characterization revealed that shifting the Ag layer from the interlayer ZnO/Ag/ZnO to the top and bottom layer Ag/ZnO/Ag reduces the absorption edge from 3.146 to 3.063 eV and the direct optical band gap from 3.24 to 3.14 eV, increasing the Urbach energy from 0.34 to 0.40 eV. These outcomes reveal that the position of the Ag layer significantly affects both structural and optical properties, suggesting potential applications in solar cells, thin-film transistors, and gas sensors.

## Abstract 2

Effect of Ag layer position in ZnO/Ag/ZnO and Ag/ZnO/Ag films was examined. Moving Ag to surface layers reduced band gap, raised Urbach energy, improved conductivity, and enabled tunable optical properties for optoelectronic and sensing uses.

## Graphical abstract

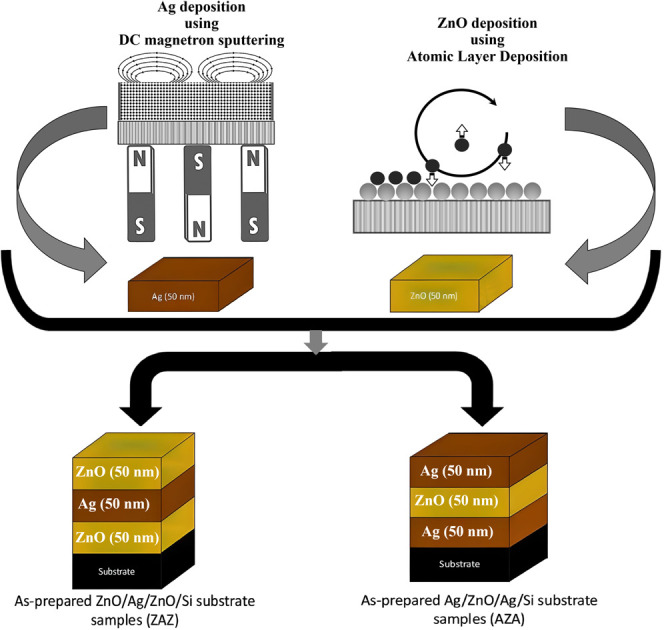

## 1. Introduction

The investigation of metal/oxide/metal and oxide/metal/oxide multilayer thin films has garnered significant interest due to their versatile applications in optoelectronic devices, transparent electrodes, and plasmonic systems. Zinc oxide (ZnO), a Group II–VI semiconductor, has garnered significant interest due to its exceptional characteristics in photovoltaics, its substantial potential to enhance the thermal and optical properties of materials, and its applications in catalysis.^1^ ZnO has been extensively studied due to its high stability, antibacterial properties, chemical effects, low cost, and non-toxicity.^2–5^ The material is also highly promising for various industrial applications, including electronic devices, electrochemical sensors, luminescence, solar cells, gas sensors, photocatalysts, surface acoustic wave filters, and electrical and optical devices, owing to its wide band gap energy and high optical transparency.^6–9^

Zinc oxide (ZnO) thin films have garnered significant attention due to their diverse applications, particularly in optoelectronics, sensing, and energy devices. ZnO thin films are widely used in optoelectronic devices such as light-emitting diodes (LEDs) and photodetectors. Integrating ZnO with other materials, such as Al_2_O_3_, through Atomic Layer Deposition (ALD) can enhance the optical dispersion parameters, which is crucial for improving the performance of devices that rely on precise light manipulation. This improvement has been demonstrated in studies where ZnO/Al_2_O_3_ thin films were fabricated, showing enhanced optical properties suitable for solar energy applications.^6^ The sensitivity of ZnO to various gases, combined with its excellent electrical properties, makes it an ideal material for gas sensors. The nanostructured forms of ZnO, such as nanowires and nanorods, increase the surface area and, consequently, the sensitivity of the sensors. These properties are especially beneficial in detecting pollutants or hazardous gases at low concentrations.^7^ Ag nanocomposites can exhibit improved sensitivity and selectivity, particularly for detecting reducing gases like hydrogen, due to the catalytic properties of Ag, which enhance the interaction between the gas molecules and the ZnO surface.^8^ ZnO thin films also find applications in energy-harvesting devices, such as piezoelectric nanogenerators. The inherent piezoelectric properties of ZnO, combined with its mechanical flexibility, enable the conversion of mechanical energy into electrical energy. This application is particularly promising for wearable devices and other applications that require low-power energy sources.^7^ ZnO is commonly used as a transparent conductive oxide in various electronic devices, including thin-film solar cells and flat-panel displays. Doping ZnO with elements like Ti has been shown to enhance its electrical conductivity further while maintaining high transparency. Precise control over doping levels, achieved through techniques such as atomic layer deposition (ALD), is critical for optimizing the performance of ZnO-based transparent conductive oxides (TCOs).^8^

Numerous studies have demonstrated the enhancement of ultraviolet-visible (UV-vis) absorption in ZnO through doping with various metals, such as Cu, Ni, Mn, and Ag, which increases the dielectric constant and thereby meets the requirements for energy storage devices.^10–13^ Among various transition metals, Ag-doped ZnO has attracted considerable attention due to its significant potential to enhance the optical properties of ZnO.^13–16^ Silver (Ag) exhibits strong absorption in the visible range and is crucial in industry and medicine due to its antibacterial activities, high thermal conductivity, and high oxidation resistance.^17–19^

ZnO/Ag-based multilayer films and nanocomposites have demonstrated significant advancements in various applications. High-quality ZnO/Ag/ZnO multilayer films, deposited *via* RF and DC sputtering, exhibit excellent optical transmittance and electrical conductivity, making them suitable for transparent conductive coatings.^20^ Optimizing the spacing of Ag nanoparticle layers in ZnO/Ag/ZnO/Ag/ZnO structures enhances the performance of UV photodetectors by increasing sensitivity and response speed.^21^ Graphene-Ag/ZnO nanocomposites, fabricated through a non-toxic solvothermal process, exhibit remarkable photocatalytic activity under visible light due to enhanced electron transport and reduced recombination rates.^22^

Research on ZnO/Ag-based thin films has also shown promising advancements in electrochemical and optoelectronic devices. A study on porous Ag-ZnO/Ag heterostructures revealed the formation of independent silver crystals within the ZnO matrix, enhancing electrochemical properties through improved charge transfer efficiency due to the ZnO/Ag Schottky contact.^23^ Another investigation demonstrated that ZnO/Ag/ZnO multilayer systems achieve around 85% transparency, making them suitable as transparent conducting electrodes for organic light-emitting diodes, with excellent optical and electrical properties maintained under mild sputtering conditions.^24^

The selection of transparent conductive oxides (TCOs), the ZnO/Ag/ZnO multilayer assembly, has emerged as a fascinating substitute due to its unique optical properties. According to a literature survey,^25^ the incorporation of transition metal “Ag” into the ZnO lattice causes the formation of new functionalities, which in turn significantly influence the material's structural and optical characteristics. As a result, the range of possible uses for ZnO can be expanded. This study investigates the structural, morphological, and optical properties of ZnO/Ag/ZnO and Ag/ZnO/Ag thin films fabricated through Atomic Layer Deposition (ALD) and DC magnetron sputtering. The article explores the fundamental effects of altering the position of the Ag layer on various optical parameters, including the absorption coefficient (*α*), extinction coefficient (*k*), optical density (OD), skin depth (*δ*), Urbach energy (*E*_U_), steepness parameter (*σ*), electron–phonon interaction (*E*_e–p_), energy gap (*E*_g_), power factor (*r*), and refractive index (*n*) of Ag-doped ZnO films.

Recent advancements in optically tunable multilayer thin films emphasize the importance of structural engineering in achieving tailored spectral responses. For instance, Ai *et al.*^26^ designed a VO_2_/Ag-based multilayer structure exhibiting phase-transition-enabled dual-band camouflage, demonstrating how smart thermochromic–plasmonic coupling can modulate infrared reflectance across multiple bands. Similarly, Chen *et al.*^27^ reported an ultra-wideband absorber combining Dirac semimetals and graphene metamaterials, achieving near-perfect absorption over a broad spectral range by leveraging interfacial coupling and photonic hybridization.

The functionality of Ag/ZnO-based multilayer thin films continues to evolve through compositional optimization and interfacial engineering. Mu *et al.*^28^ synthesized mesocrystalline ZnO “twin-cake” structures decorated with CdS and Cu nanoparticles, achieving remarkable degradation of pharmaceutical contaminants under visible light. The enhanced performance was attributed to tunable oxygen vacancies and synergistic effects at the heterointerfaces. Dai *et al.*^29^ reported that Mn doping and surface modification of ZnS nanoparticles significantly enhance charge separation, band alignment, and light absorption in Bi_2_MoO_6_-based systems, resulting in a high degradation efficiency of tetracycline under solar irradiation. Sun *et al.*^30^ demonstrated that hollow nanospheres with a core–shell–shell architecture [Au@CdZnS@MnO_2_] achieve significantly improved CO_2_ photoreduction performance through electron configuration modulation. The inclusion of Au nanoparticles within the CdZnS shell induces a strong localized surface plasmon resonance (LSPR), promoting charge separation and extending light absorption into the visible and near-infrared regions. Thus, the recent studies underscore the growing role of metal-dielectric multilayers and interface design. This motivated us to explore the Ag/ZnO/Ag configuration in our work by tailoring optical behavior for advanced optoelectronic and camouflage applications. This study provides a comprehensive analysis of how the position of the Ag layer in ZnO/Ag/ZnO (ZAZ) and Ag/ZnO/Ag (AZA) thin films influences their structural, morphological, and optical properties. By employing Atomic Layer Deposition (ALD) for ZnO and Direct Current (DC) magnetron sputtering for Ag, the research uniquely explores how shifting the Ag layer's location affects parameters such as crystallite size, dislocation density, surface roughness, absorption edge, and energy gap. This detailed comparison between ZAZ and AZA thin films highlights new insights into how structural modifications can tailor the properties of these materials for various applications. Consequently, this research aims to elucidate the effective role of Ag layer doping in ZnO and its applications, encompassing ultraviolet light-emitting devices, ultraviolet photodetectors, solar cells, and thin-film transistors.

The paper is divided into three key sections. The Experimental section outlines the detailed methodology for preparing and analyzing ZnO/Ag/ZnO (ZAZ) and Ag/ZnO/Ag (AZA) thin films. The Results and discussion section is subdivided into multiple subsections, each exploring different facets of these thin films. Finally, the Conclusion section highlights this study's significant findings and insights.

## 2. Experimental

Two types of sandwich structure samples were deposited by ALD for the ZnO layers and by DC magnetron sputtering for the Ag layers with a constant ZnO and Ag thickness of 50 nm and a full nominal thickness of 150 nm for each sample on glass substrates at a deposition temperature of 100 °C in thermal mode. This deposition temperature was chosen based on prior studies that have shown optimal structural and optical outcomes at this temperature. Mahdizadeh *et al.*^8^ demonstrated that Ag:ZnO nanocomposites deposited at or near 100 °C exhibit superior uniformity, reduced roughness, and well-defined grain growth compared to films deposited at higher temperatures, where Ag agglomeration becomes more pronounced. Moreover, Atomic Layer Deposition (ALD) of ZnO at low thermal budgets (∼100 °C) helps maintain the structural integrity of thermally sensitive substrates, such as glass, while still enabling high film quality and tunable optical band gaps.^6^ Maintaining this lower deposition temperature also minimizes unwanted interdiffusion or phase formation at ZnO/Ag interfaces, preserving the distinct multilayer structure critical to this study. Thus, the selection of 100 °C balances the need for process compatibility, layer purity, and desirable material properties in both ZAZ and AZA films.

**Fig. 1:**
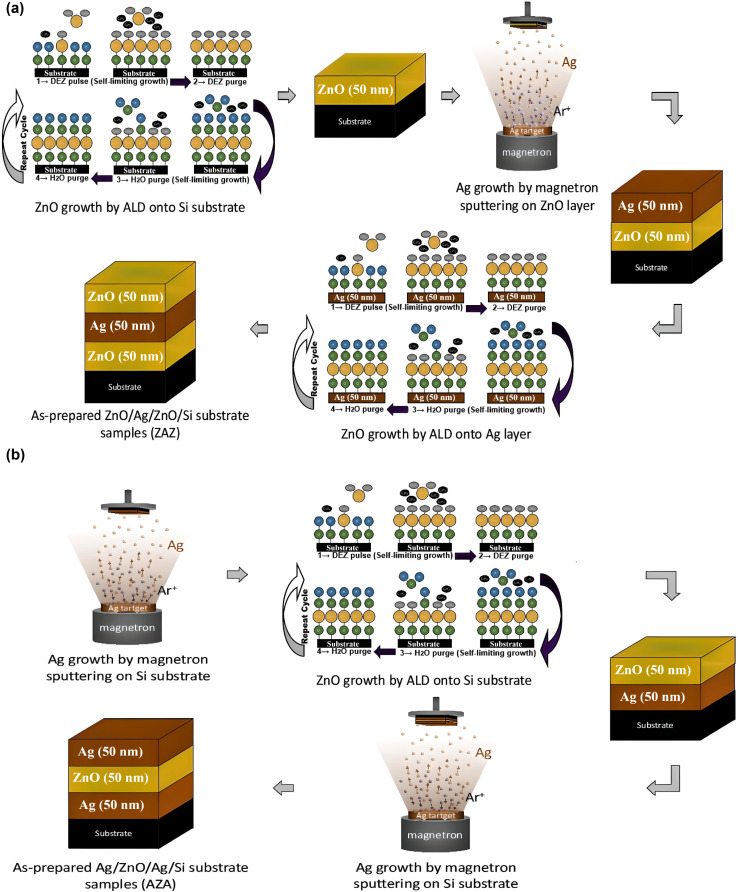
(a) Schematic of the important steps adopted in the present study to prepare ZAZ samples using the ALD and DC magnetron sputtering techniques. First, ZnO is grown on a substrate by ALD, then Ag is deposited on the ZnO by DC magnetron sputtering, and at the end, ZnO is grown on the Ag layer by ALD. (b)Schematic of the important steps adopted in the present study to prepare AZA samples using the ALD and DC magnetron sputtering techniques. First, Ag is deposited on a substrate by DC magnetron sputtering, then ZnO is grown on the Ag by ALD, and at the end, Ag is deposited on the ZnO layer by DC magnetron sputtering.

The two thin film samples prepared of ZnO (50 nm)/Ag (50 nm)/ZnO (50 nm) and Ag (50 nm)/ZnO (50 nm)/Ag (50 nm), will be labeled as ZAZ and AZA, respectively. The schematic drawings of the ZAZ and AZA sandwich structures are shown in Fig. 1.

The tri-layer structure was fabricated through a carefully controlled deposition process:

(i) Zinc oxide (ZnO) thin films were deposited onto substrates *via* atomic layer deposition (ALD) using a Beneq TFS-200 system. During the process, a chamber pressure of approximately 1 mbar was maintained in the reaction zone and 9 mbar in the main compartment. Diethylzinc (DEZ) and water served as the chemical precursors. The deposition was carried out in thermal mode at a substrate temperature of 100 °C. A total of 305 ALD cycles were performed, with each cycle consisting of a 300 ms pulse of both DEZ and H_2_O, followed by a 3 second nitrogen purge. The cycle count was optimized based on spectroscopic ellipsometry data acquired with a Semilab SE-2000.

(ii) Silver (Ag) films were subsequently deposited at ambient temperature using DC magnetron sputtering. The base vacuum pressure within the sputtering chamber was maintained below 3 × 10^−7^ mbar. During deposition, the Ar pressure (99.999%) (under dynamic flow) was 7 × 10^−3^ mbar. The purity of Ag was 99.99%. The deposition rate was calibrated using an Ambios XP-1 profilometer. First, we pump down the preparation chamber to achieve the base pressure. The base pressure affects the cleanliness of the layers. The lower the base pressure, the less contamination from the chamber can build up in the layers. Once the base pressure is reached, high-purity argon is injected into the chamber (dynamic flow), which is necessary to create the Ar plasma. The Ar ions in the plasma are then accelerated towards the target (Ag in our case). The Ar ions bombard the target, knocking atoms out of the target material (Ag in our case), which are then deposited on the substrate. The pressure of the Ar gas is, therefore, critical for generating the plasma, and the purity of the Ar gas affects the quality of the plasma (*i.e.*, the purity of the grown layers). Grain size can be influenced by the substrate's temperature, for example. This also affects the surface roughness. Increasing the temperature will increase the grain size and decrease the surface roughness. However, other parameters, such as the DC power, influence the film properties. These parameters must be optimized with great accuracy in each magnetron sputtering system, as they are critical for reproducibility.

X-ray reflectivity (XRR) (Rigaku smart Lab) and grazing incidence X-ray diffraction (GIXRD) were used to identify the structures of ZAZ and AZA. The GIXRD and XRR data were fitted with Rigaku's Smart Lab Studio II software. Using an energy-dispersive X-ray spectrophotometer (EDX) (Shimadzu diffractometer type XRD 6000) and a scanning electron microscope (SEM) (JEOL JSM Model 5600), the elemental composition of ZAZ and AZA was investigated. The optical absorption and transmittance spectra were measured using a double-beam UV-vis spectrophotometer (SP, V-570, JASCO, Japan).

## 3. Results and discussion

### 3.1 Structural modification

**Fig. 2:**
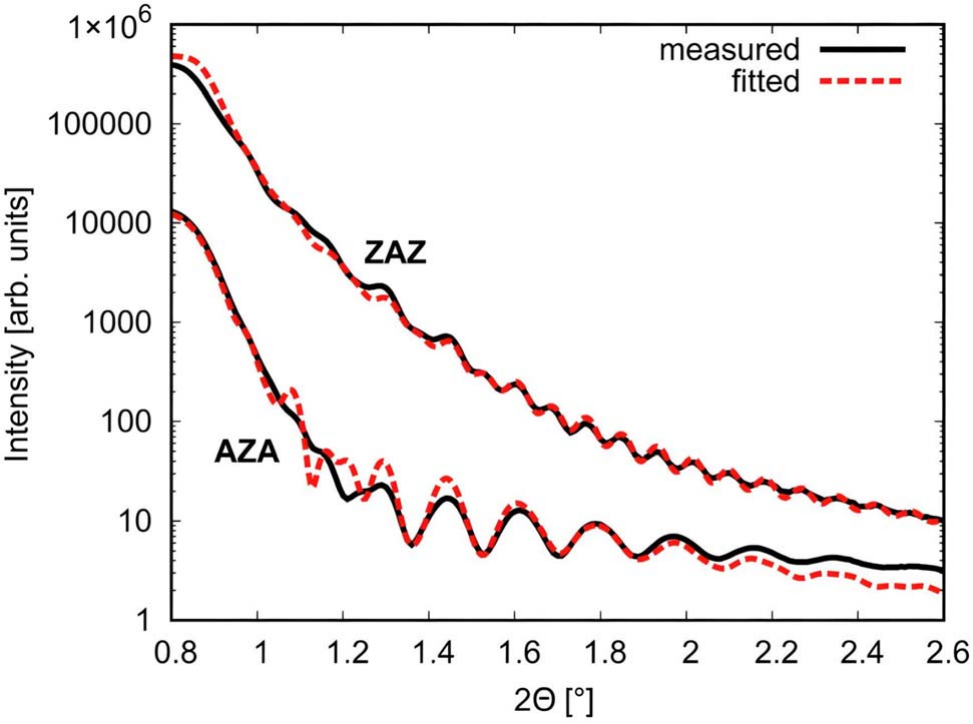
XRR plots for ZAZ and AZA thin films respectively.

**Fig. 3:**
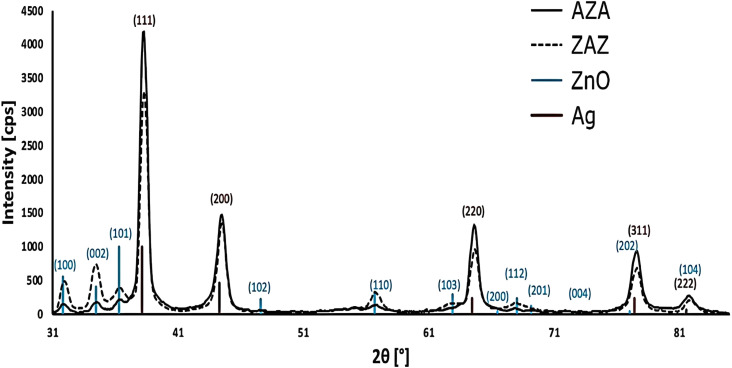
GIXRD diffraction patterns of ZAZ and AZA thin films. Grazing incidence X-ray diffraction (GIXRD) patterns of ZAZ and AZA thin films, showing peaks corresponding to hexagonal wurtzite ZnO (2*θ* values: 31.59°, 34.38°, 36.24°) and face-centred cubic Ag (2*θ* values: 38.11°, 44.28°, 64.46°, 77.1°, and 81.1°). No impurity phases were observed. Structural differences in crystallite size and peak intensity suggest changes in layer morphology with varying Ag positions.

The structural parameters, including thickness, mass density, and roughness, of ZAZ and AZA thin films were evaluated using XRR fitting patterns shown in Fig. 2 and presented in Table 1. The resulting curve (see Fig. 2) presents the best fit between the measured and predicted values. The use of GIXRD and XRR measurements provides direct confirmation of the multilayer architecture of the fabricated thin films, which is confirmed from GIXRD patterns for both ZAZ and AZA configurations (Fig. 2) and clearly show distinct diffraction peaks corresponding to the crystalline Ag (fcc) and ZnO (wurtzite) phases, indicating successful sequential deposition of the individual layers. Additionally, the XRR profiles (Fig. 3) confirm the presence of layered thin films through detectable variations in reflectivity intensity, consistent with multiple electron density transitions along the film depth. The profiles support the successful formation of stratified ZAZ and AZA architectures with compositional contrast between the ZnO and Ag layers.

**Table 1:** Values of thickness, mass density, crystallite size, dislocation density, and roughness for ZAZ and AZA thin films

Sample	Thickness (nm)			Density (g cm^−3^)	Crystallite size (nm)	Dislocation density (nm^−2^)	Roughness (nm)
ZAZ	45.1 (Zn)	50.0 (Ag)	47.2 (Zn)	5.7 (ZnO) 10.5 (Ag) 5.7 (ZnO)	15	4.2 × 10^−3^	8.3
AZA	49.8 (Ag)	44.9 (Zn)	49.5 (Ag)	10.5 (Ag) 5.7 (ZnO) 10.5 (Ag)	17	3.4 × 10^−3^	2.9

As indicated in Fig. 3, the structural patterns of the ZAZ and AZA thin films using GIXRD indicate the hexagonal wurtzite structure of the investigated samples. The layer thicknesses are close to the nominal values of 50 nm each. The physical density for ZAZ and AZA is also presented in Table 1. The characteristic sharp diffraction peaks of ZnO were located at 2*θ* = 31.59°, 34.38°, and 36.24° corresponding to the peaks (100), (002), and (101), accompanied by other weak reflections.^31^ These observations agree with previous research.^32,33^ The presence of certain randomly oriented grains was confirmed by weak diffraction peaks corresponding to ZnO. Broad and pronounced peaks were also seen in addition to these reflections. These peaks are positioned at 2*θ* values = 38.11°, 44.28°, 64.46°, 77°.1°, and 81.1°, representing planes (111), (200), (220), (311), (222) caused by elemental growth of silver (Ag), and are found to be in a good agreement with those in the literature.^34^ No other impurity phases were found in the diffraction pattern of our investigated ZAZ and AZA thin film samples. The black vertical lines show the positions of the Ag peaks (taken from the ICCD PDF database), while the blue vertical lines correspond to the positions of the ZnO peaks. The length of the lines is proportional to their intensities in the database. The solid and dashed black curves show the diffraction patterns measured on the AZA and ZAZ samples.

Structural parameters such as average crystallite size and dislocation density for ZAZ and AZA thin films were evaluated from the GIXRD patterns and are presented in Table 1. The average crystallite size (*D*) value of the ZAZ and AZA thin films in terms of Bragg angle *θ* (*i.e.*, the angle at which the X-ray diffraction peak is observed) was determined using the recognized Debye–Scherrer equation:^34^1
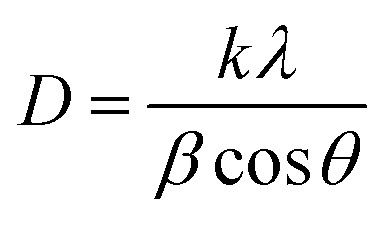
In eqn (1), *k* is the shape factor (dimensionless, typically around 0.9, but can vary depending on the shape of the crystallites), *λ* is the wavelength of the X-ray radiation, and *β* is the full width at half maximum (FWHM) of the X-ray diffraction peak (in radians).

The dislocation density (*δ*) presents the degree of crystallinity. The (*δ*) can be calculated with a known (*D*) by using the equation:^35^2
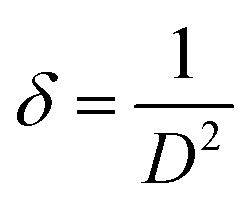


The results of (*D*) and (*δ*) are summarized in Table 1. As shown, the dislocation density decreases with increasing crystallite size, indicating a lower number of lattice imperfections when the position of the Ag interlayer is changed. For further investigation, we used a spectroscopic ellipsometer (model SE-2000; manufacturer Semilab, Hungary) and profilometer measurements to determine the average surface roughness of ZAZ and AZA thin films, as tabulated in Table 1. The Semilab SE-2000 Spectroscopic Ellipsometer is a variable-angle ellipsometer featuring an automatic mapping stage, a parallel beam, and a microspot. It operates with the new operating and analysis software (SAM and SEA). The state-of-the-art SE-2000 system, featuring a rotating compensator configuration, can achieve high-accuracy ellipsometric measurements in the wavelength range of 190 nm to 25 μm. It supports fully automated angles of incidence from 10° to 90° (having an accuracy of ∼0.01°) and provides fast data acquisition. Data analysis and modelling were performed using Semilab's Spectroscopic Ellipsometry Analyzer (SEA) and Spectroscopic Analysis Module (SAM) software.

Typically, the surface roughness of thin films increases with increasing crystallite size; however, our results show the opposite trend. The multilayer thin film sample is a particular case because the fabrication technique is also responsible for various trends in surface roughness. The growth in crystallite size with a reduction in roughness arises from boundaries between grains, resulting in more scattering when comparing ZAZ with AZA. The observed link between surface roughness and crystallite size was strongly clarified, and how Ag is incorporated into ZnO and *vice versa* affects the optical properties, as will be seen later.

Table 1 shows that the crystallite sizes for the ZAZ and AZA thin films are 15 nm and 17 nm, respectively. These values are comparable to those reported in similar studies using ZnO/Ag systems. For example, in a study by Zheng *et al.*, ZnO/Ag nanostructures prepared *via* magnetron sputtering exhibited crystallite sizes ranging from 15.45 nm to 29.73 nm, depending on the thickness of the Ag interlayer.^36^ Similarly, Kuriakose *et al.* synthesized Ag/ZnO nanocomposites *via* a wet chemical method and reported crystallite sizes ranging from 8 to 20 nm.^37^ Another study by Pham *et al.* on Ag/ZnO nanoparticles prepared for photocatalytic applications indicated that the crystallite size of pure ZnO was approximately 20.1 nm. The Ag/ZnO nanocomposite was slightly reduced to 19.6 nm.^38^ Additionally, Primo *et al.* reported larger crystallite sizes (32.5–33.9 nm) for Ag/ZnO nanocomposites synthesized for antibacterial applications, attributed to differences in synthesis routes.^39^ The slight differences in crystallite sizes across these studies can be attributed to variations in deposition techniques, substrate temperatures, and post-deposition annealing processes.

### 3.2 Surface morphological analysis

**Fig. 4:**
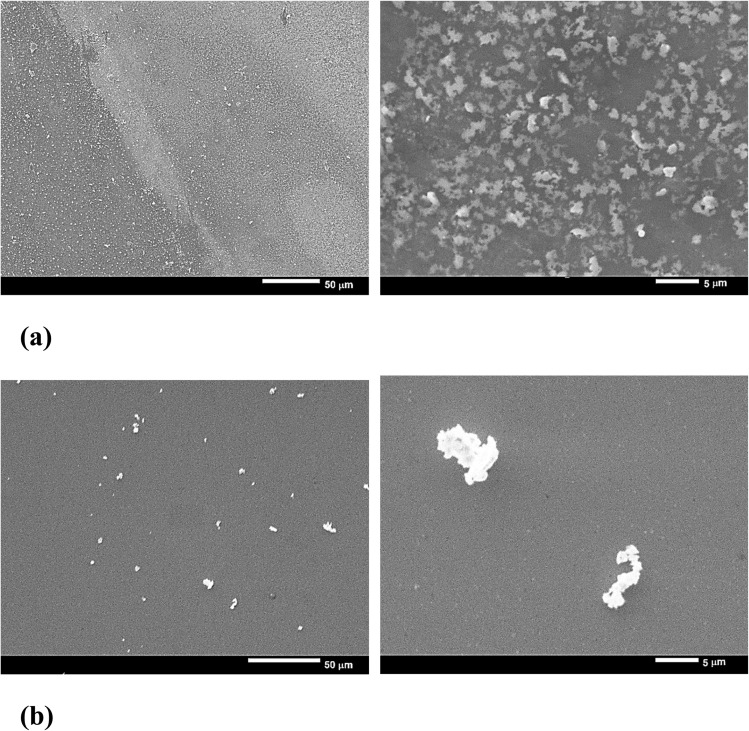
SEM images of (a) ZAZ thin films and (b) AZA thin films at two different magnifications.

Fig. 4 shows the SEM images at two different magnifications for the thin film samples of ZAZ and AZA. The improvement in grain growth was observed when the ZAZ thin films were compared with the AZA thin films. The ZAZ thin film sample exhibits a smooth surface covered with small grains, whereas changing the layer sequence to AZA yields a granular structure as shown in the SEM images.

The SEM images of ZnO/Ag/ZnO thin films in Fig. 4(a) show a relatively uniform surface with some visible textures and patterns. There are larger-scale surface features and variations, indicating some degree of surface roughness and texture. The distribution of features appears to be relatively homogeneous across the sample. When we increase the resolution from 50 μm to 5 μm, numerous small, granular structures are visible, indicating the presence of fine particles or clusters. The surface appears densely packed with these granular features, suggesting a high surface area. The SEM image of the Ag/ZnO/Ag thin films at 50 μm shows a much sparser distribution of surface features compared to the ZnO/Ag/ZnO films. It also indicates the presence of fewer visible surface defects or particles, indicating a smoother and more uniform surface. At comparatively high resolution (5 μm), the surface morphology shows distinct, larger particle clusters. These clusters are less densely packed compared to the granular structures observed in the ZnO/Ag/ZnO films. The individual clusters are more pronounced and isolated, indicating a different deposition or growth mechanism.

**Fig. 5:**
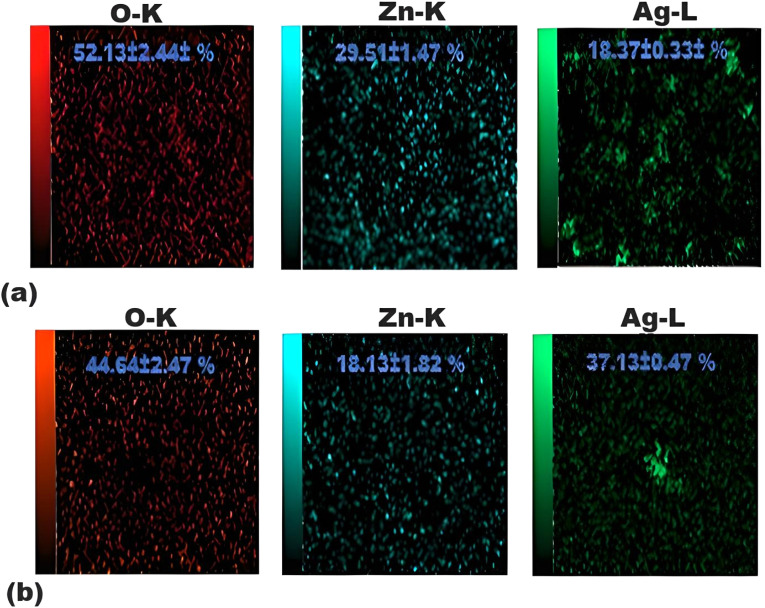
The elemental maps of (a) ZAZ thin films and (b) AZA thin films corresponding to their EDS spectra.

Elemental mapping is a powerful technique for analyzing and presenting information on the spatial distribution of elements in any sample more effectively. This is typically performed in an SEM image using energy-dispersive spectroscopy (EDS). High-resolution images of the area of interest were collected, along with the EDS data. The results are presented in Fig. 5 for both samples, along with corresponding elemental maps. Visualizing the spatial distribution of ZnO and Ag in the ZAZ and AZA thin film samples confirms the existence of the ZnO and Ag elements.

### 3.3 Absorption analysis

**Fig. 6:**
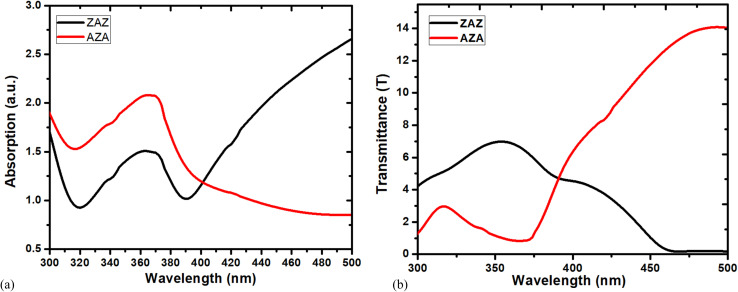
Absorption (a) and transmittance spectra (b) of ZAZ and AZA thin films.

Absorbance values offer excellent sensitivity in measurements, particularly for low concentrations, where the difference between transmittance values becomes noticeable, making it more challenging to distinguish any differences. Hence, it is convenient to use absorbance measurements rather than transmittance because absorbance is proportional to the concentration of the analyte, whereas % transmittance is not. Fig. 6(a) shows the significant enhancement of UV-vis absorption spectra for ZAZ and AZA thin films. The absorption spectra of the ZAZ and AZA thin films show significant differences in the UV region. The enhanced UV absorption peak at around 360 nm is attributed to the fundamental band gap transitions. The increased absorption in the AZA film compared to the ZAZ film can be explained by its higher refractive index and denser structure, as indicated in Table 1. Furthermore, the presence of larger silver clusters in AZA films, as shown by SEM analysis (Fig. 4), likely leads to enhanced localized surface plasmon resonance effects, contributing to increased UV absorption.

According to Fig. 6, the structural results and the absorption variation in the UV region support each other. It is well known that absorbance and transmission have an inverse relationship, so a higher absorbance indicates less transmittance. The absorbance and transmittance spectra in Fig. 6 for ZAZ and AZA thin films confirm the inverse relation mentioned previously. The growth in grain size enhances the structural and optical characteristics by increasing the transmittance, as confirmed by comparing Fig. 6(b) with the results of the crystallite size of ZAZ and AZA presented in Table 1.

**Fig. 7:**
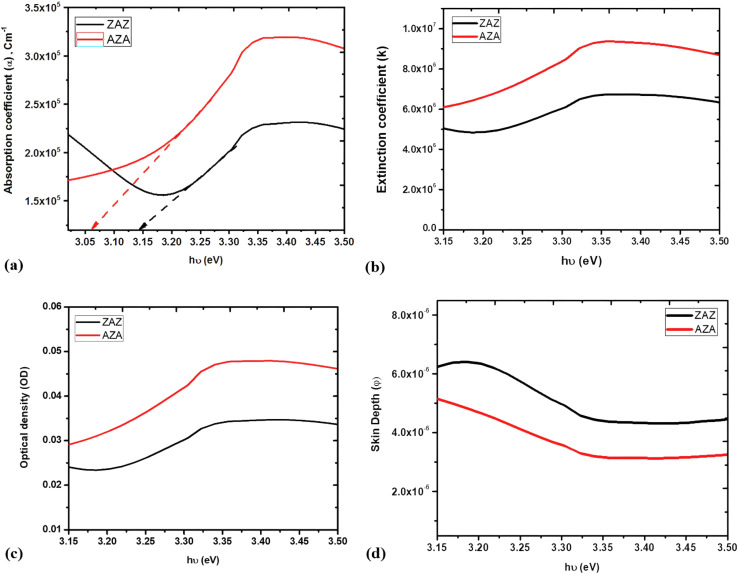
Plots of (a) *α versus hν*, (b) *k versus hν*, (c) OD *versus hν*, and (d) skin depth *versus hν* for ZAZ and AZA thin films.

A straightforward formula, *α* = 2.303*A*/*t*, can be used to calculate the absorption coefficient (*α*) from the absorbance. In this formula, “*t*” stands for thin film thickness and “*A*” for absorption. The above equation was used to plot (*α*) *versus* (*hν*) of ZAZ and AZA thin films as seen in Fig. 7(a). Fig. 7(a) and Table 2 illustrate that the absorption coefficient (*α*) of ZAZ is lower than that of AZA. The absorption edge values have been estimated by extending the straight-line segments of the plots to intercept the energy axis. It is evident that the AZA sample shifts the absorption edge toward lower photon energies relative to the ZAZ sample. This downward shift, from 3.146 eV to 3.063 eV, suggests alterations in the electronic band structure, potentially due to the emergence of new localized states within the band gap. This would suggest a decrease in the band gap energy (*E*_g_).

**Table 2:** Values of various optical parameters [*E*_U_, *σ*, *E*_e–p_, *E*_g_, and *n*_av_] for ZAZ and AZA thin films

Sample	Absorption edge (eV)	*E*_U_ (eV)	*σ* (eV)	*E*_e–p_ (eV)	*E*_g_ (eV)	Refractive index				*n*_average_
						*n*_1_	*n*_2_	*n*_3_	*n*_4_	
ZAZ	3.146	0.34	6.8 × 10^−4^	9.8	3.24	2.32	2.33	2.30	2.25	2.30
AZA	3.063	0.40	5.8 × 10^−4^	11.3	3.12	2.34	2.36	2.33	2.29	2.33

To examine the structural alterations of the ZAZ and AZA thin film samples that are being studied, the value of the extinction coefficient (*k*) can also be obtained from the formula of the absorption coefficient *α*:^40^3
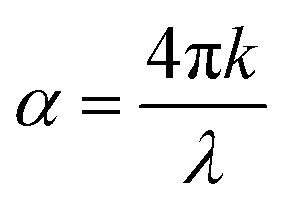


The extinction coefficient depends on the structure and wavelength of the incident light, and it is greater when the absorption is more intense. Fig. 7(b) shows the determined values of (*k*) for ZAZ and AZA.

The behaviour of *k* shown in Fig. 7(b) supports the behaviour of absorption (*A*) as shown in Fig. 6(a), where the absorption of AZA is greater than that of ZAZ in both mentioned parameters. The optical density (OD) is a convenient tool for describing the transmittance of light or any other electromagnetic radiation. The values of OD for the thin films of ZAZ and AZA samples are governed by the relation OD = *αt*, where “*t*” is the film thickness. In this work, the thickness for ZAZ = 142.3 nm, while the thickness for AZA = 144.2 nm. Fig. 7(c) presents the plots of OD for AZA and ZAZ against photon energy (*hν*).

The optical density remains constant between 3.30 and 3.5 eV, and then increases with a steep gradient above 3.2 eV. It is also clear that the variation of (OD) confirms the similarity with the behaviour of (*α*) for AZA and ZAZ thin films [see Fig. 7(b)]. The higher OD values of AZA than those of ZAZ indicate that light travels more slowly through the AZA thin film than through ZAZ. The observed optical density (OD) behaviour correlates well with the measured surface roughness values, as detailed in Table 1. Moreover, the inverse relationship between optical density and surface roughness can be attributed to density primarily influencing total reflectance, whereas surface roughness predominantly affects diffuse reflectance. Furthermore, the skin depth or penetration depth (*ϕ*) is linked to the absorption coefficient and is proportional to the square root of resistivity. The skin depth of the ZAZ and AZA thin films was evaluated by using the formula:^41^4
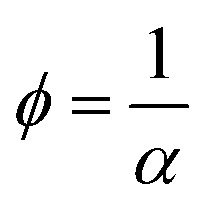


The plots of *ϕ versus hν* for ZAZ and AZA thin films are depicted in Fig. 7(d). It was found that the skin depth of ZAZ and AZA decreased with the increase in photon energy, whereas ZAZ exhibits the highest skin depth, indicating that the conductivity of AZA is higher than that of ZAZ.

The Urbach energy (*E*_U_) is a critical parameter that quantifies the absorption associated with electronic transitions between the valence band tail and conduction band states. The parameter *α* characterizes the width of the exponential tail in the absorption edge resulting from structural disorder, defect states, and electron–phonon interactions, and it exhibits an exponential dependence on photon energy as described by the empirical Urbach relation:^42^5
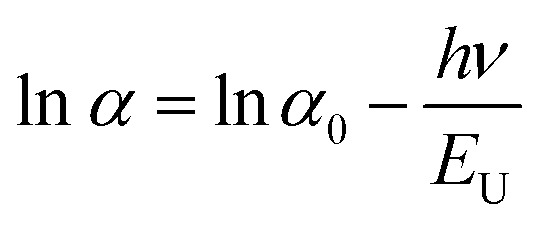
Here, *α*_0_ represents the absorption coefficient at the extrapolated zero photon energy. Furthermore, we would like to note that “zero photon energy” is not physically realizable, as photons with zero energy do not exist. However, in the context of the Urbach relation, extrapolating to zero energy provides a reference point for the absorption process. *α*_0_ reflects the density of localized states near the band edges. A higher *α*_0_ indicates a greater number of defect- or disorder-induced states contributing to absorption at low photon energies. The value of *α*_0_ is influenced by the material's microstructure (*e.g.*, grain boundaries, dislocations, or amorphous regions). For instance, materials with more disorder tend to have higher *α*_0_, implying stronger sub-bandgap absorption. Thus, at very low photon energies (*i.e.*, near zero), the absorption coefficient approaches the hypothetical value *α*_0_. Therefore, *α*_0_ serves as a baseline from which absorption increases exponentially with photon energy.

The parameter *E*_U_ is known as Urbach energy, which characterizes the width of the tail of the absorption edge and is related to the degree of disorder in the material. As shown in Fig. 6(b) the ZAZ thin films exhibit higher transmittance in the UV region due to their lower density and smaller crystallite size, resulting in fewer scattering centres and defects. The better-defined band edges of ZAZ films, indicated by the steeper slope of the absorption edge, are consistent with their lower Urbach energy (Table 2).

**Fig. 8:**
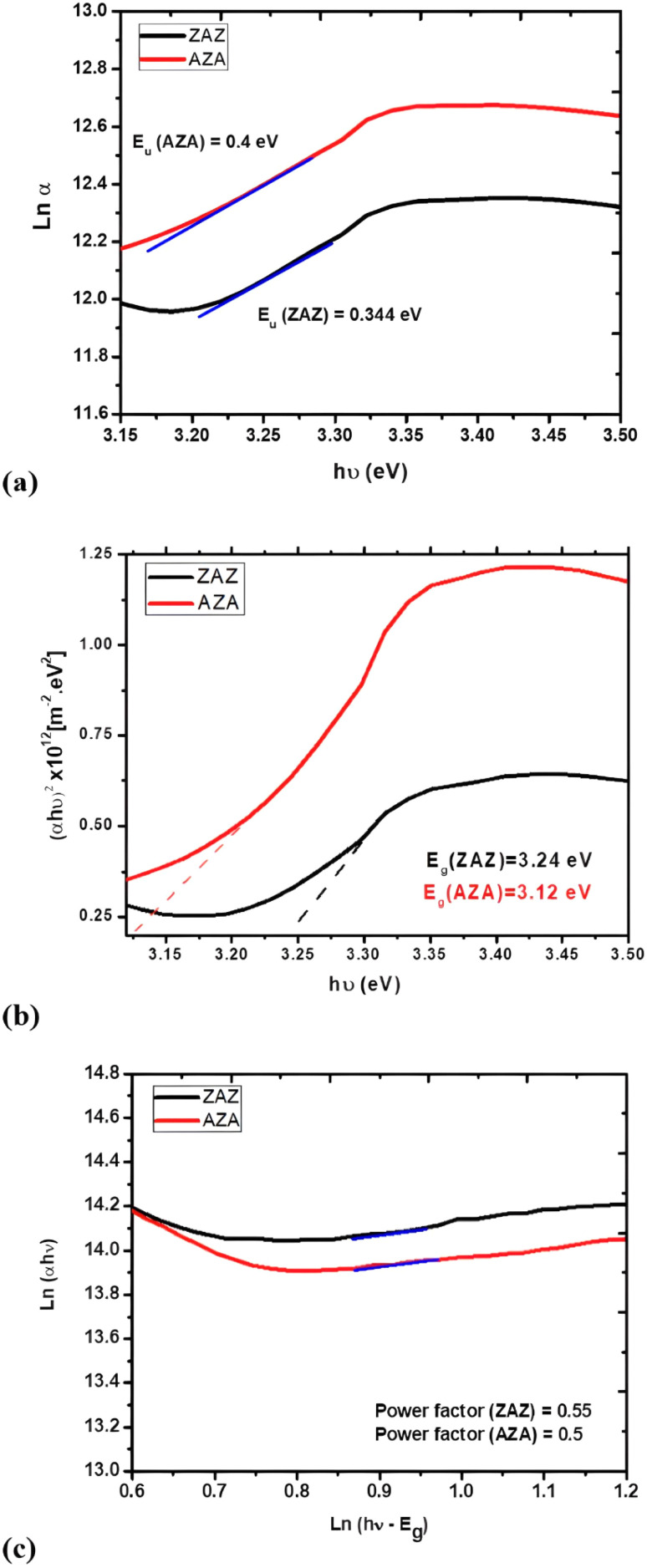
Plots of (a) ln *α versus hν*, (b) (*αhν*)^2^*versus hν*, and (c) ln(*αhν*) *versus hν* for ZAZ and AZA thin films.

The plots of ln *α versus hν* for ZAZ and AZA thin films are shown in Fig. 8(a). From this figure, the *E*_U_ values can be estimated. The creation of the defects in the band structure is confirmed by the increase in *E*_U_ for AZA compared to ZAZ (as observed in Table 2). In the band structure of amorphous materials, the density and kind of flaws and disorder are intimately connected with the Urbach energy. Higher Urbach energy values indicate more severe disorders and a higher concentration of defects, which broadens the exponential tail of the absorption edge. The magnitude of this exponential tail is expressed in terms of the Urbach energy. A higher Urbach energy indicates a broader tail due to more substantial disorder and a higher density of localized states. The steepness parameter (*σ*) can provide us with information on the expansion of the absorption edge that occurs due to the interaction of electrons and phonons. The steepness parameter can be calculated as a function of temperature using the following equation:^43^6
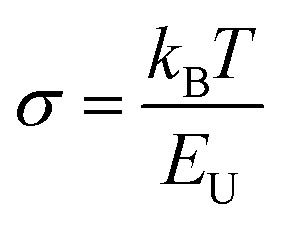
In eqn (6), *k*_B_ is the Boltzmann constant, and *T* is the absolute temperature. The values of *σ* for ZAZ and AZA are listed in Table 2.

The electron–phonon interaction strength, or *E*_e–p_, is correlated with the *σ* and can be calculated using the equation^44^ below:7
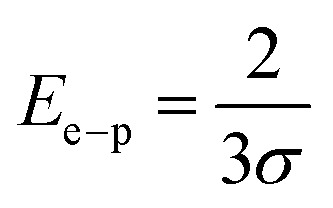


The estimated value of the electron–phonon interaction strength for ZAZ and AZA thin films is tabulated in Table 2. The data reveal that both the *E*_U_ and *E*_e–p_ exhibit an increase, while a decrease in *σ* occurs as the position of the Ag layer in the ZAZ and AZA thin films is altered. This phenomenon can be attributed to the variation in the ionicity and anion valence of the ZAZ and AZA thin film samples. The optical energy gap *E*_g_ of the ZAZ and AZA thin films can be determined from the optical absorption spectra using the Tauc's energy exponential relation:^45^

8 *αhν* = *A*(*hν* − *E*_g_)^*r*^.

In eqn (8), *A* represents a constant, while the exponent *r* takes on values of 0.5 or 2 for direct and indirect transitions, respectively. Our analysis revealed that the optimal fit occurs at *r* = 0.5, corresponding to an allowed direct transition, as depicted in Fig. 8(b). The calculated values of (*E*_g_) for the two films under investigation are indicated in Table 2.

The results indicate that the position of the Ag layer in the ZAZ and AZA thin films deepens the band tail, which extends into the gap, consequently increasing the value of *E*_U_.

Furthermore, the reduction in band gap energy (*E*_g_) is not solely due to crystallite size variation, but also arises from the creation of defect levels and increased carrier–phonon interactions, which broaden the energy distribution at the band edges. This is consistent with the observed increase in the electron–phonon interaction parameter (*E*_e–p_) in AZA samples. Jalal *et al.*^52^ also demonstrated that Ag inclusion in doped ZnO films leads to a similar narrowing of the band gap and enhancement of sub-gap absorption due to local field enhancement and band tailing effects. This enriched understanding supports the role of Ag placement in modulating the degree of disorder and energy level distribution in multilayer thin films.

To clearly elucidate the nature of electronic transitions (direct or indirect) in the investigated thin film samples, the optical energy gap must first be determined, followed by the power factor *ρ* using the equation.^41^

9 ln(*αhν*) = ln *B* + *ρ*(*hν* − *E*_g_)

In eqn (9), *B* is a constant. This process can show the type of transition by plotting ln(*hν* − *E*_g_) *versus* ln(*hν*) as seen in Fig. 8(c).

The estimated values of the transition power factor for ZAZ and AZA are approximately equal, *ρ* ≅ 0.5. This process confirms the existence of the direct power transition that has been determined from eqn (9) and Fig. 8(b) as well. As shown in Table 2, the optical band gap (*E*_g_) decreases from 3.24 eV to 3.12 eV when the Ag layer is relocated from the middle layer to either the top or bottom layer. This shift suggests that altering the Ag layer's position leads to increased energy state dispersion, promoting the formation of tail states in the band structure. Furthermore, evaluating additional optical parameters, such as the refractive index (*n*), is essential when selecting suitable materials for optoelectronic devices.

The parameter *n* is derived from various equations, contingent upon the value of the optical energy gap (*E*_g_), as defined by the following relationships:^46–49^

10 *n*_(1)_^4^*E*_g_ = 95 eV

11
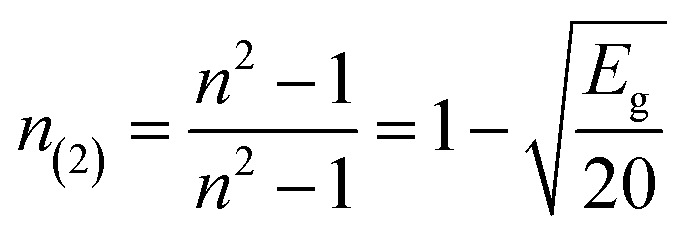

12 *n*_(3)_ = *K*(*E*_g_)^*c*^

13
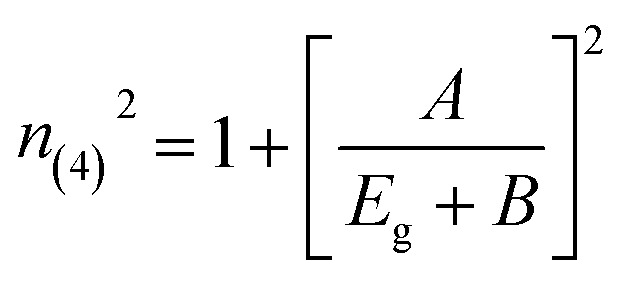


The values of the constants *k*, *c*, *A*, and *B* used in eqn (12) and (13) are 3.3668, −0.32234, 13.6 eV, and 3.47 eV, respectively.^47,48^

Table 2 presents the mean refractive index values (*n*_av_) calculated using the four aforementioned models. A higher *n*_av_ in AZA corresponds to an increase in ZAZ and a greater concentration of charge carriers. Consequently, AZA exhibits a higher optical density compared to ZAZ, as illustrated in Fig. 7(c), where optical density is directly related to the refractive index. Additionally, an inverse relationship is established between the optical band gap (*E*_g_) and crystallite size.

When the frequency of the material is considered in Gaussian units, it reveals essential aspects of its optical behavior, notably through optical conductivity (*σ*_opt_). This parameter characterizes the interaction of the material with electromagnetic waves, offering insights into charge carrier transport and associated energy loss mechanisms.

To calculate the optical conductivity, (*σ*_opt_) the following relation is used:^50^14
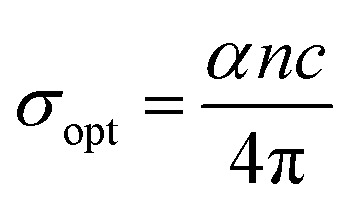
Here, *c* is the speed of light in a vacuum (*c* = 3 × 10^10^ cm s^−1^).

The electrical conductivity *σ*_ele_ in terms of optical conductivity *σ*_opt_ can be expressed as:^50^15
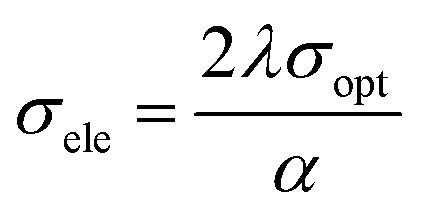


**Fig. 9:**
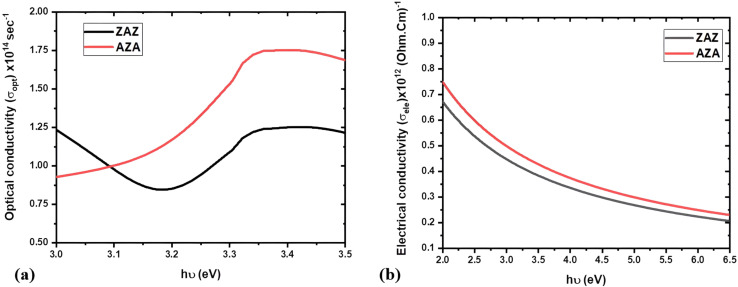
Plots of (a) *σ*_opt_*versus hν* and (b) *σ*_ele_*versus hν* for ZAZ and AZA thin films.

The plots of *σ*_opt_ and *σ*_ele_ as a function of photon energy (*hν*) for both samples are shown in Fig. 9(a) and (b). These properties are directly related to the absorption coefficient (*α*), as both conductivities rely on the interaction of photons with the material. The AZA film exhibits higher optical conductivity beyond 3.1 eV compared to the ZAZ film. This can be attributed to the AZA sample's denser structure and enhanced refractive index, as seen in Table 2. The position of the Ag layer in AZA likely promotes stronger localized surface plasmon resonance (LSPR) effects, which enhance the interaction between light and free electrons, increasing the optical conductivity. Similarly, the AZA film exhibits higher electrical conductivity due to its lower dislocation density and smoother surface (Table 1). The improved structural integrity minimizes scattering and defects, facilitating better charge carrier mobility.

Considering Tables 1 and 2, the comparison between ZnO/Ag/ZnO (ZAZ) and Ag/ZnO/Ag (AZA) thin films reveals distinct differences in their structural and optical properties, which impact their applicability and usefulness. ZAZ thin films exhibit a lower mass density (5.7 g cm^−3^) than AZA (10.5 g cm^−3^), indicating a denser structure for AZA. The ZAZ films have a smaller crystallite size (15 nm) and a higher dislocation density (4.2 × 10^3^ nm^−2^), resulting in greater surface roughness (8.3 nm) compared to AZA's smoother surface (2.9 nm). This finding is consistent with those of Cullity,^33^ which indicate that grain boundary density is inversely related to structural disorder.

Optically, ZAZ films display a higher absorption edge (3.146 eV) and lower Urbach energy (0.34 eV) compared to AZA, which shows values of 3.063 eV and 0.40 eV, respectively. The lower Urbach energy for ZAZ films than that of AZA films indicates reduced disorder. This trend can be further understood by considering the effect of Ag layer positioning on the local atomic environment and electron–phonon interactions. The AZA structure, having Ag on both outer surfaces, likely experiences stronger interface strain and defect formation at the ZnO/Ag boundaries, which in turn creates localized states within the band gap. These states contribute to the exponential tail of absorption, resulting in a higher Urbach energy. Belahssen *et al.*^42^ noted that a lower Urbach energy correlates with fewer localized states and defects near the band edges, resulting in sharper absorption edges. The higher optical bandgap of ZAZ films (3.24 eV) compared to AZA films (3.12 eV) further supports the existence of better-defined electronic states. Studies by Yu *et al.*^17^ and Sahu *et al.*^20^ indicate that materials with a higher bandgap generally exhibit reduced structural disorder, as fewer sub-bandgap defect states are available for absorption. This suggests that ZAZ films have more defined band edges and less structural disorder. The ZAZ film also exhibits slightly lower refractive indices across all measured wavelengths, indicating improved transparency. Recent studies continue to affirm the relevance of Ag–ZnO systems in optoelectronic and functional material applications. For instance, Alzahran *et al.* reported efficient Ag/ZnO-based systems for water treatment applications, highlighting the role of Ag incorporation in improving optical absorbance and contaminant removal under visible light irradiation.^51^

Jalal *et al.* reported that Ag nanoparticle coating on W-doped ZnO films significantly narrows the band gap and enhances UV photodetector responsivity due to localized surface plasmon resonance (LSPR) and interface-state modulation.^52^ In another study, Ahmed *et al.*^53^ demonstrated that both the ZnO film thickness and substrate temperature critically influence the crystallinity and optical transparency of ALD-grown films, which directly supports our choice of a 100 °C growth temperature. Furthermore, Patwa *et al.*^54^ synthesized ZnO/CuO/Ag nanocomposites *via* a coprecipitation method, demonstrating enhanced structural uniformity and broad-spectrum photocatalytic activity due to synergistic effects between Ag and the ZnO–CuO matrix.

The structural and optical distinctions between ZAZ and AZA thin films have meaningful implications for their targeted applications. The ZAZ configuration, with its higher transmittance, sharper absorption edge, and lower Urbach energy, is particularly well-suited for optoelectronic applications where minimal defect-induced scattering and high transparency are critical, such as in ultraviolet-transparent window layers in solar cells or photodetectors. Conversely, the AZA films, which exhibit enhanced optical and electrical conductivity along with a higher refractive index and optical density, are more appropriate for plasmonic or thermally reflective coatings, transparent conducting electrodes, and gas sensing devices where strong light–matter interaction and carrier mobility are advantageous. Future work can focus on tuning the Ag layer thickness and exploring asymmetric layer designs (*e.g.*, ZnO/Ag/Al-doped ZnO) to modulate these optical responses further. Additionally, hybrid structures incorporating VO_2_ or graphene in conjunction with ZnO/Ag layers could be developed for dual-band absorption or dynamic infrared control, as inspired by recent developments in tunable multilayer nanostructures.^26^ These directions would not only enhance the performance but also expand the functionality of such layered oxide-metal systems. Thus, the denser and smoother AZA films may be more suitable for applications requiring robust structural integrity and higher refractive indices, such as thin film transistors and gas sensors. Conversely, the ZAZ films, with their higher transparency and lower structural disorder, are potentially more advantageous for optoelectronic applications, such as solar cells, where efficient light absorption and minimal scattering are critical.

## 4. Conclusions

ALD and DC sputtering were used to form ZAZ and AZA thin films on glass substrates at 100 °C. The influence of changing the position of the Ag layer on the structural, morphological, and optical properties was studied. The GIXRD patterns indicate that ZAZ and AZA are polycrystalline with a modulated crystal structure and that the surface properties are improved by changing the Ag layer from ZAZ to AZA. The SEM images show well-defined grains for the ZAZ thin film, while a granular feature is seen for the AZA thin film. The distribution of the content in the thin film samples under study was confirmed through elemental mapping. The SEM images of the ZAZ and AZA thin films reveal that the ZnO/Ag/ZnO thin films exhibit a more uniform and densely packed surface morphology, characterized by numerous small granular structures. In contrast, the Ag/ZnO/Ag thin films show a sparser distribution of larger clusters, suggesting a less dense surface structure. Thus, ZnO/Ag/ZnO films have a densely packed, granular structure, whereas Ag/ZnO/Ag films display a sparser distribution with larger isolated clusters.

The investigation revealed that changing the position of the Ag layer from ZAZ to AZA reflected an increase in Urbach energy, refractive index, and optical density. Analysis of our results confirms the inverse relationship between crystallite size and energy gap, dislocation density, and surface roughness, as well as the relationship between the energy gap and the refractive index. In light of the above-mentioned results, it is suggested that the position of the Ag layer plays a significant role in governing the subject mentioned above and would be beneficial in several applications when added to ZnO. In future work, angle-resolved optical studies, such as spectroscopic ellipsometry or UV-vis measurements at varying incidence angles, will reveal the angular dependence of transmittance, reflectance, and absorption in ZAZ and AZA multilayer films. Such studies would provide deeper insight into their potential use in optoelectronic and photonic devices where directional light behavior is critical.

## Author contributions

S. S. Fouad: supervision, writing final draft, conceptualization. L. I. Soliman: sharing in writing – original draft, formal analysis. M. Nabil, M. E. Sayed, and N. F. Osman: plotting graphs, formal analysis. János J. Tomán and E. Baradács: preparing thin film samples, formal analysis. Zoltán Erdélyi: supervision, preparing thin film samples, formal analysis, conceptualization. Neeraj Mehta: writing and editing the final draft.

## Conflicts of interest

The authors declare that they have no known competing financial interests or personal relationships that could have appeared to influence the work reported in this paper.

## Data Availability

The authors declare that the data supporting the findings of this study are available within the paper.
